# Transperitoneal Mini-Laparoscopic Pyeloplasty and Concomitant Ureteroscopy-Assisted Pyelolithotomy for Ureteropelvic Junction Obstruction Complicated by Renal Caliceal Stones

**DOI:** 10.1371/journal.pone.0055026

**Published:** 2013-01-09

**Authors:** Zhi Chen, Peng Zhou, Zhong-Qing Yang, Yang Li, Yan-Cheng Luo, Yao He, Nan-Nan Li, Chao-Qun Xie, Chen Lai, Xiao-Long Fang, Xiang Chen

**Affiliations:** 1 Department of Urology, Xiangya Hospital, Central South University, Changsha, Hunan, China; 2 Department of General Surgery, Xiangya Hospital, Central South University, Changsha, Hunan, China; The University of Manchester, United Kingdom

## Abstract

**Objective:**

To present our experience of combining transperitoneal mini-laparoscopic pyeloplasty (mini-LP) and concomitant ureteroscopy-assisted pyelolithotomy (U-P) for ureteropelvic junction obstruction (UPJO) complicated by renal caliceal stones in the same session.

**Methods:**

Between May 2007 and December 2011, mini-LP and concomitant U-P was performed in nine patients with UPJO and ipsilateral renal caliceal stones. Stone location and burden were preoperatively assessed. After pyelotomy with appropriate length (about 4 mm), a 16-Fr catheter sheath replaced the uppermost or lowermost laparoscopic trocar and was introduced directly into the renal pelvis under the guidance of a guide wire and laparoscopic vision. A 7.5F rigid ureteroscopy passed through the catheter sheath into the plevis. Intracorporeal lithotripsy and/or pressure irrigation via a pump was used for caliceal stone removal. Subsequently, laparoscopic pyeloplasty was performed in a standard fashion. Postoperative imaging was assessed.

**Results:**

The calculi sizes ranged from 2 to 11 mm (mean, 7.1 mm) and an average of 3 stones per patient was removed (range, 1 to 6 stones). Complete stone clearance confirmed by postoperative imaging was achieved in all patients. Mean operative time was 210 minutes, and estimated blood loss was 20 mL. Mean hospital stay was 5 days (4–7). Stent was removed after 4–8 weeks. No intraoperative or postoperative complications were noted during a mean follow-up of 18.5 months (range, 6 to 24 months).

**Conclusions:**

Mini-LP and concomitant U-P are simple and effective alternatives for the simultaneous management of UPJO complicated by coexisting ipsilateral renal caliceal stones.

## Introduction

Ureteropelvic junction obstruction (UPJO) complicated by the presence of ipsilateral calculus disease, especially renal caliceal calculus, poses a technically challenging situation for the urologist. Although the surgical treatment of UPJ obstruction associated with renal calculi has evolved significantly over the past 2 decades [1], [2], [3], [4], [5], [6], [7], [8], [9], [10], [11], [12], [13], [14], [15], there is still therapeutic controversy regarding the ideal minimally invasive management. In the present study, we report a minimally invasive and reproducible technique that greatly facilitates the surgical treatment of this morbidity, using a 7.5F rigid ureteroscopy during mini-laparoscopic pyeloplasty for caliceal stone removal.

## Patients and Methods

### Patients

**Figure 1:**
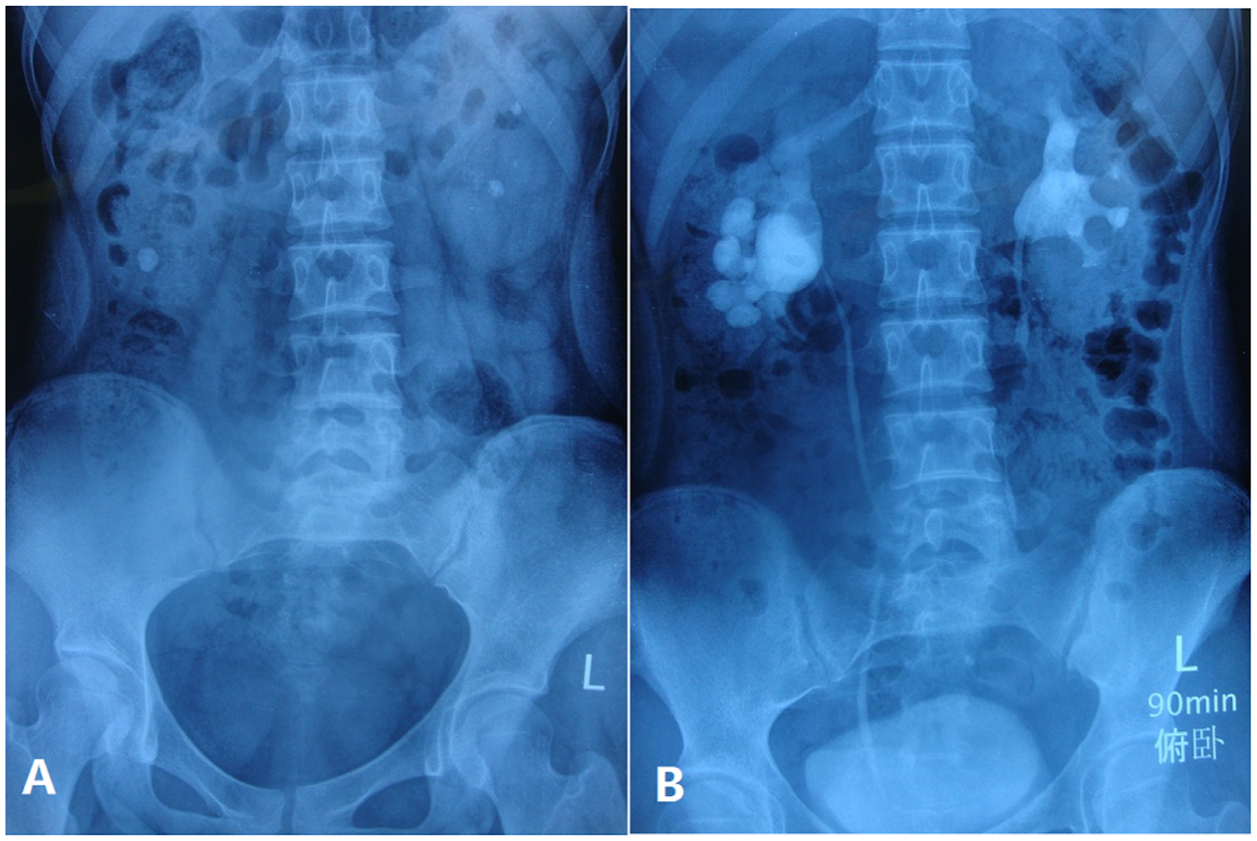
Preoperative assessment for stone location and burden. (A) Preoperative kidney–ureter–bladder X-ray. (B) Preoperative intravenous urography.

Between May 2007 and December 2011, nine patients with UPJO and ipsilateral renal caliceal stones underwent transperitoneal mini-laparoscopic pyeloplasty (mini-LP) and concomitant ureteroscopy-assisted pyelolithotomy (U-P) at our institution. This study obtained ethics approval from the ethics committee at Xiangya Hospital, Central South University, Changsha, Hunan Province, China. Also, we obtained informed consent from the adult participants or from the parents of the children participants in our study. The informed consent was written and specified in the operative consent. The patients included 5 men and 4 women, with an average age of 26.1 years (range, 16–42 years). Of the 9 patients, 7 had UPJO on the left side and 2 on the right side. All patients presented with mild to moderate flank pain. No patients had undergone previous abdominal surgery in our series. Stone location and burden were preoperatively assessed. **(** **Figure 1** **)** A combination of diuretic renal scans, intravenous urography (IVU), kidney–ureter–bladder X-ray and/or CT was postoperatively performed to assess the drainage pattern and to evaluate any residual stones.

### Operative Technique

**Figure 2:**
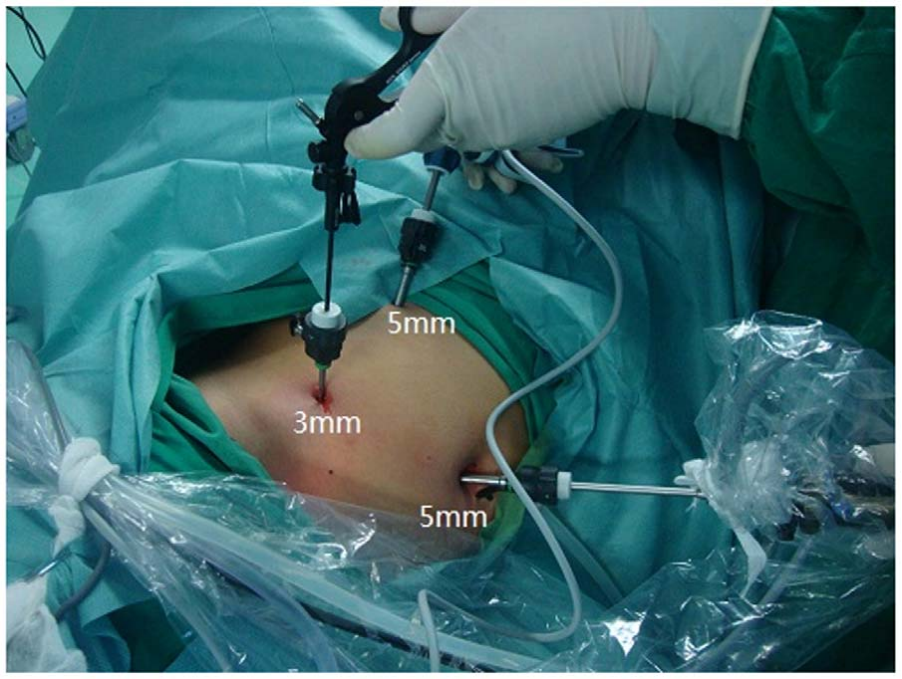
Port-placement for transperitoneal mini-laparoscopic pyeloplasty. A 5-mm port was placed infraumbilically for the camera (A) and one 5-mm port and one 3-mm port in the midclavicular line ipsilaterally (B and C). (right side).

Under general endotracheal anesthesia, patients were placed in a 45° lateral decubitus position. We used the three-port tranperitoneal approach. The pneumoperitoneum was obtained by carbon dioxide insufflation through a Veress needle at 12–14 mm Hg of intraabdominal pressure. A 5-mm port was placed infraumbilically for the camera and one 5-mm port and one 3-mm port in the midclavicular line ipsilaterally under direct vision using the laparoscope. **(** **Figure 2** **)**.

**Figure 3:**
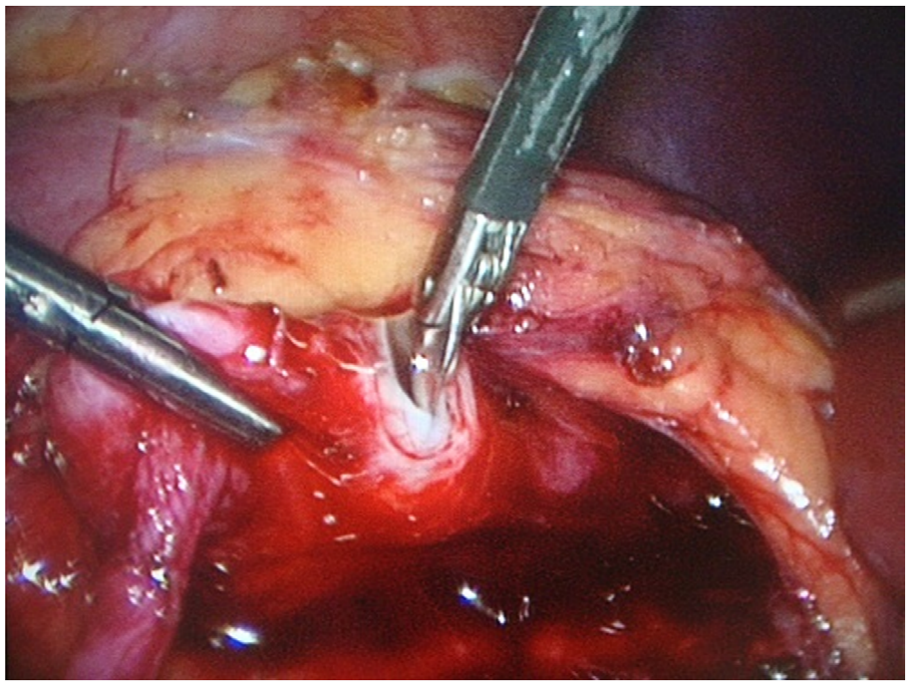
Performance of pyelotomy. A pyelotomy was preformed with appropriate length (about 4 mm) to achieve the pyeloplasty.

**Figure 4:**
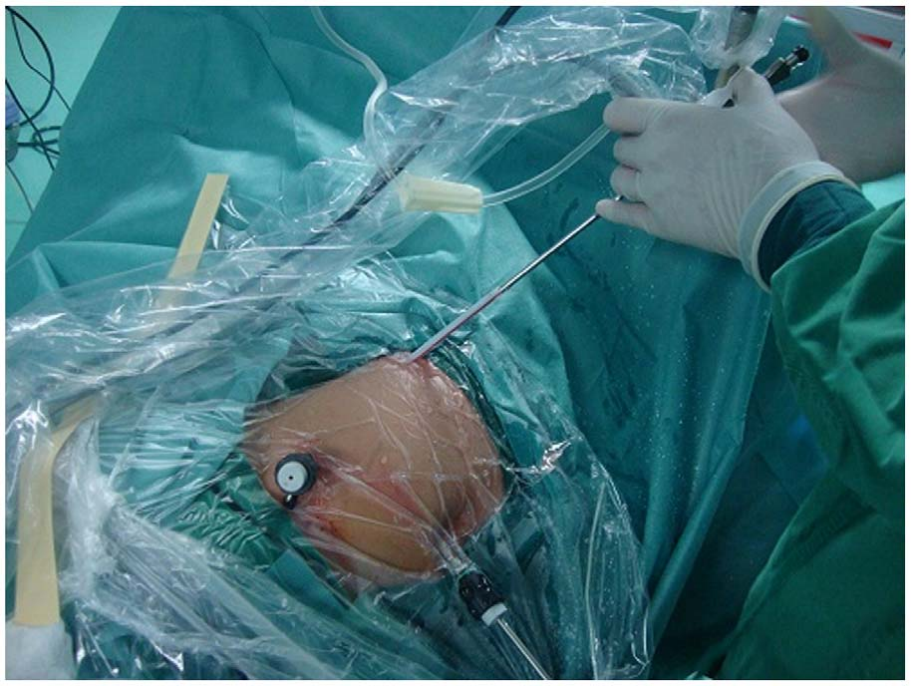
Introduction of rigid ureteroscopy. A 7.5F rigid ureteroscopy was introduced through the catheter sheath into the plevis.

**Figure 5:**
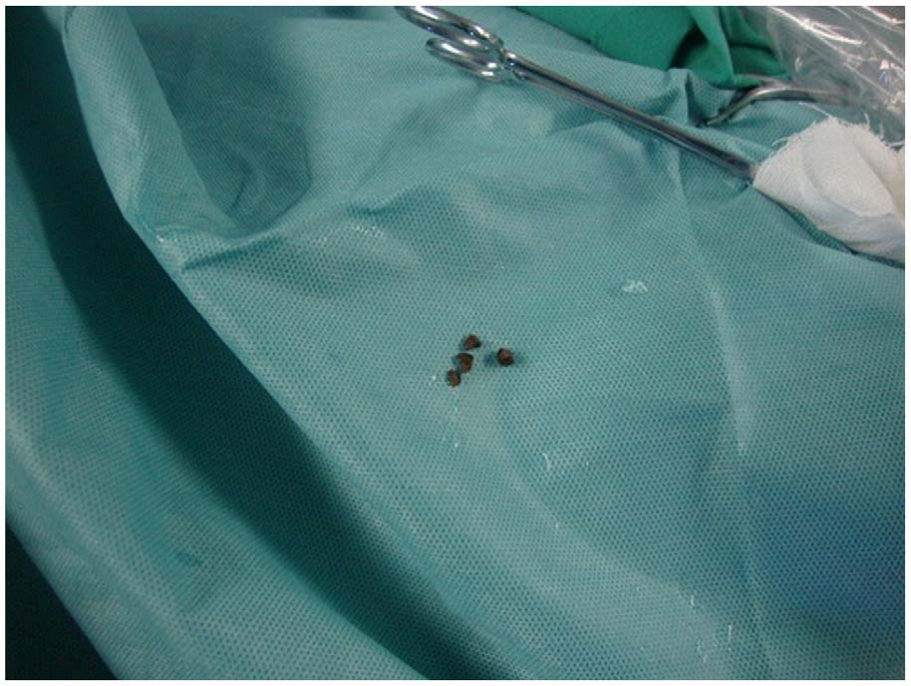
Renal caliceal calculus. Intracorporeal lithotripsy was performed and the calculus was flushed out.

**Figure 6:**
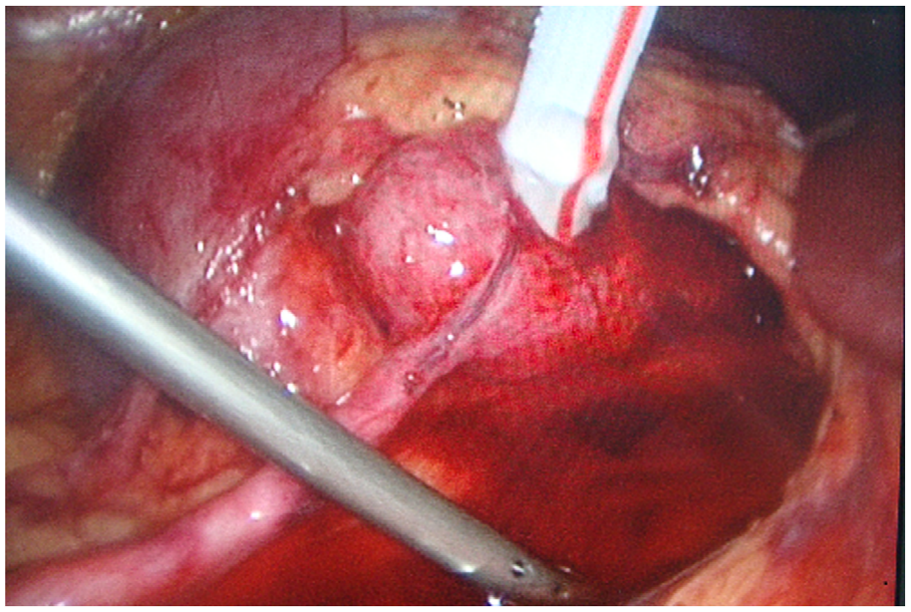
Aspiration of irrigant. The irrigant was aspirated by the suction probe placed just below the pyelotomy through the other laparoscopic port.

After the laparoscopic instruments were introduced, the line of Toldt was incised to reflect the colon medially and the kidney was exposed using standard laparoscopic techniques. The dilated renal pelvis was easily identified and carefully dissected down to the proximal ureter. Any crossing vessel at the UPJ was carefully dissected free from the UPJ if present. Whether a crossing vessel was required for transposition was determined based on individual anatomy. Then a pyelotomy was preformed with appropriate length (about 4 mm) to achieve the pyeloplasty. **(** **Figure 3** **)** A guide wire was passed through the uppermost (for middle or lower caliceal calculus) or lowermost (for middle or upper caliceal calculus ) laparoscopic trocar and pyelotomy, directly into the renal pelvis under laparoscopic vision. The trocar was removed and a 16-Fr catheter sheath with a core was introduced under the guidance of the guide wire. After the guide wire and core was removed, a 7.5F rigid ureteroscopy was introduced through the catheter sheath into the plevis and the abdominal pressure was routinely decreased to 6–8 mmHg. **(** **Figure 4** **)** If the calculus was big, intracorporeal lithotripsy was routinely performed. **(** **Figure 5** **)** Otherwise, pressure irrigation via a pump was used to flush out the small calculus. The irrigant was aspirated by the suction probe placed just below the pyelotomy through the other laparoscopic port. **(** **Figure 6** **)**.

Subsequently, laparoscopic pyeloplasty was performed in a standard fashion. In brief, the pelvis was partly divided using a “cold” scissors through the lowermost trocar, from the most dependent part, cephalad toward the pelvis. The most lateral extent of the pelvis was left attached to the ureter for traction, which serves as a handle that can stabilize the ureter during subsequently spatulating, suturing and excising. The scissors was introduced through the uppermost trocar and the ureter was easily spatulated laterally. This change greatly facilitates this step because the axis of the scissors was almost in line with the ureteral axis.

**Figure 7:**
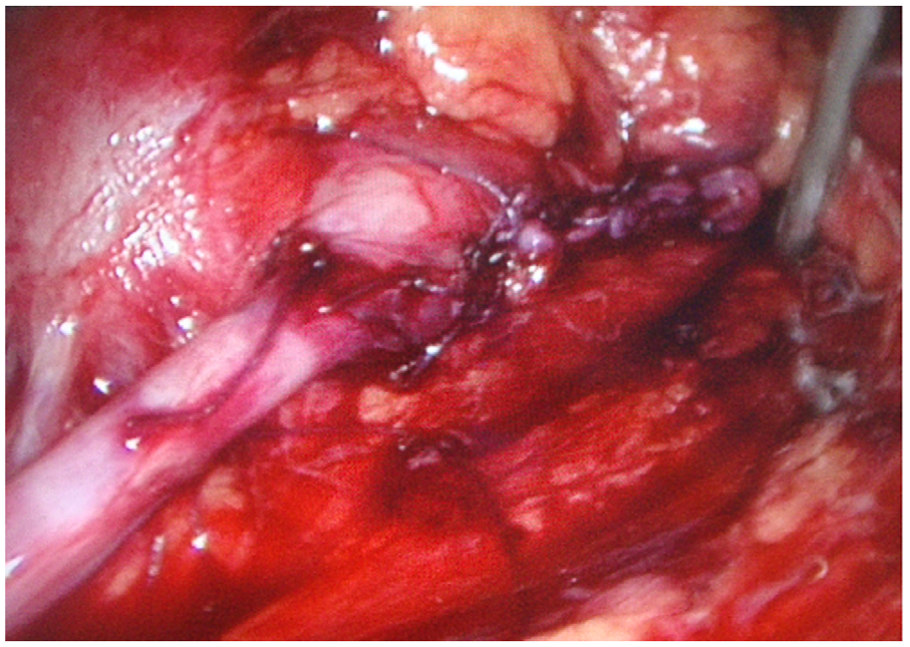
Ureteropelvic anastomosis. Anastomosis was completed using 4-0 Vicryl (Ethicon) sutures.

A 4-0 Vicryl (Ethicon) suture was used for anastomosis. The most dependent part of the pyelotomy was sutured to the apex of the spatulated ureter. The UPJ and the redundant pelvis were excised. After completion of the posterior anastomosis with an interrupted suture and the remaining pyelotomy anastomosis with a running suture, a Double-J stent was placed in an antegrade manner. Subsequently, the anterior wall is closed with an interrupted suture. **(** **Figure 7** **)** A drain was placed through the lowermost port.

## Results

The calculi sizes ranged from 2 to 11 mm (mean, 7.1 mm) and an average of 3 stones per patient was removed (range, 1 to 6 stones). Mean operative time was 210 minutes, and estimated blood loss was 20 mL. In our initial 2 cases, a 16-Fr catheter sheath with a core was directly passed through the previous abdominal incision and pyelotomy into the renal pelvis without the guidance of a guide wire. Pelvis wall tear occurred during the procedure in the case 2. Thereafter, to decrease the risk of pelvis wall tear, we started the practice of routinely using a guide wire for the guidance of a 16-Fr catheter sheath with a core in the remaining 7 patients and experienced no that complication. In the case 5, the pyelotomy was unexpectedly preformed with a relatively longer length (about 8 mm). The opened up renal pelvis were partly closed by suturing with a 4-0 Vicryl suture (case 2) or holding the cut ends with the help of laparoscopic graspers (case 5) to keep the system distended for better visualization during ureteroscopy and minimize the chances of stone loss. In the case 6, we encountered problem in localizing the stones during the procedure, which was confirmed to located in the anterior middle calyces by preoperative CT. We struggled for a while before we realized that further mobilizing the kidney could significantly improve visualization angle of the ureteroscopy. These stones was successfully found and flushed out.

Complete stone clearance confirmed by postoperative imaging was achieved in all patients. Mean hospital stay was 5 days (4–7). Stent was removed after 4–8 weeks. No major intraoperative or postoperative complications were noted during a mean follow-up of 18.5 months (range, 6 to 24 months).

## Discussion

Over the past 25 years, minimally invasive procedures (endopyelotomy and laparoscopic pyeloplasty) have replaced open pyeloplasty as the standard of care for UPJO. [5] However, presence of coexisting upper urinary tract stones in the patients with UPJO presents a therapeutic dilemma to the urologist. The surgical treatment for this morbidity has traditionally been open pyelolithotomy with pyeloplasty, but it is associated with considerable morbidity and protracted convalescence. Generally, the current concept in dealing with UPJO concomitant with renal stones could be that while taking into consideration the underlying anatomy, comorbidities, and patient preference, the surgeon can choose the most suitable operation to correct anatomical abnormality of UPJO that led to stasis and stone formation and concomitantly completely clear stones through minimally invasive surgery if surgery is indicated.

Laparoscopic pyeloplasty (LP) with concomitant pyelolithotomy [1], [6], [7], [8], [9], [10], [11], percutaneous nephrolithotomy (PNL) with endopyelotomy [3], [12], [13], [14], [15] or robotic assisted laparoscopic pyeloplasty (RALP) with concomitant pyelolithotomy [4] and PNL with LP [2] have been reported for treating UPJO concomitant with renal stones. Each of these methods has its own limitations. The overall success rates for patients treated with endopyelotomy are lower than those treated with open or laparoscopic pyeloplasty. [16] Several factors, including the presence of a crossing vessel, length of narrowing, preoperative split renal function, degree of hydronephrosis and the presence of contrast extravasation at the time of surgery, influence success rates for endopyelotomy, [17] Nonetheless, in a highly selective patient population, endopyelotomy can achieve equivalent success rates compared with laparoscopic pyeloplasty. [18], [19] Therefore, PNL with endopyelotomy remains an effective alternative treatment for certain patients with UPJO complicated by renal stones, and can be easily performed by most urologists Although laparoscopic or assisted laparoscopic pyeloplasty produces success rates (87.5% to 100%) equivalent to open pyeloplasty with a 10% to 15% higher success rate when compared to endopyelotomy [20], [21], [22], [23], it needs a steep learning curve or advanced laparoscopic training. In addition, in order to remove the stones, use of flexible nephroscopy during LP or RALP could also be very technically demanding. Furthermore, PNL with LP could be very cumbersome and time-consuming for requiring repositioning. Moreover, bleeding could occur and the nephrostomy catheter could exodus during laparoscopic manipulation. Guidelines on urolithiasis of European Association of Urology (EAU) recommends that simultaneous stone removal be performed with percutaneous or transureteral endopyelotomy and open or laparoscopic reconstructive surgery for patients with UPJO.

In our centre, LP has been the first choice for patients with UPJO. Because flexible nephroscopy are not covered by medical insurance in China for its expensive price and high deterioration, the patients have to bear these charges themselves. Therefore, we developed our own surgical technique to suit this technically challenging procedure. The key point of our technique is the use of the 7.5F rigid ureteroscopy. The option of the laparoscopic trocar, through which the ureteroscopy was introduced, was determined by the location of the caliceal stones. Generally, the uppermost trocar was for lower caliceal calculus and lowermost for upper caliceal calculus. For middle caliceal calculus, the both trocars seem reasonable. We believed that these modifications can achieve the advantages of flexible nephroscopy, but lower technically challenge was needed. In addition, proper preoperative identification of the stone-bearing calix was very important for successful stone extraction. [1] Furthermore, in our procedure, the introduction of 5-mm laparoscopy and 7.5F rigid ureteroscopy only need two 5-mm incisions and one 3-mm incision. Compared to the previous report [4], in which two 12-mm ports and two 8-mm ports were used, the surgical morbidity could be further reduced.

The length of pyelotomy before introducing the ureteroscopy should be appropriate (about 4 mm), which could decrease the spillage of the irrigant and maintain a certain pressure of the colleting system. This could be helpful to flush out the small calculus. In addition, the spillage of the irrigant can be aspirated by the suction probe placed just below the pyelotomy through the other laparoscopic port.

Mini-Laparoscopic pyeloplasty was successful in relieving obstruction in all patients (100%) in our series with a mean follow-up of 18.5 months and the stone-free rate was 100%. Although the present series included only 9 patients, we showed clearly that transperitoneal LP and concomitant U-P is technically feasible, safe and can be accomplished reasonably quickly and patients with UPJO and ipsilateral renal calculi can undergo simultaneous treatment of both conditions and expect good results in regard to resolution of obstruction and complete stone clearance. Our results were similar to the data reported in previous series. [1], [2], [4], [13].

Stein et al. [1] reported a 5-mm atraumatic laparoscopic bowel grasper or lexible nephroscopy were used for extraction of renal caliceal stones during LP with concomitant pyelolithotomy. With a mean follow-up of 5.4 months, the obstruction relieving rate and stone-free rate was 93.3% (14/15) and 80% (12/15), respectively. Mean operative time was 174 minutes. Atug et al. [4] reported the use of robotic graspers in one patient and flexible nephroscopy in seven patients for pyelolithotomy during RALP. With a mean follow-up of 12.3 months, both the obstruction relieving rate and stone-free rate were 100%. Mean operative time was 275.8 minutes. Agarwal and coworkers [2] reported their experience of combining PNL with LP for UPJO with renal stones in the same session (8 cases) or staged manner (2 cases). Mean operative time was 234 minutes. At 6 months patients are stone free on ultrasound and show good drainage on renal scan. Berkman and colleagues [13] retrospectively reviewed patient data for all endoscopic pyeloplasties performed at a single institution over 10 years. Forty-one patients underwent simultaneous nephrolithotomy for ipsilateral renal calculi. Complete lithotomy was confirmed visually and with live fluoroscopy in all cases except those with radiolucent stones. [13] For 30 patients with>12 months of follow-up, the overall obstruction relieving rate was 90% in the nephrolithotomy and endopyelotomy group. Their operative time was not reported.

In the present study, the mean operative time was lower than that reported by Agarwal et al [2]and Atug et al [4] (210 vs 234 and 275.8 minutes, respectively). The reduction of the mean operative time may be explained by several reasons. First, our series only included the patients with UPJO and ipsilateral renal caliceal stones and the calculi sizes were small (2 to 11 mm). Therefore, our present series could be relatively easily managed. Second, with the use of the 7.5F rigid ureteroscopy, our technique could be less technical demanding. In addition, our mean operative time was higher than reported by Stein et al. [1], who used a laparoscopic grasper or pressure irrigation via a pump for removing stones in the majority of cases and intraoperative flexible nephroscopy was only used if the afore-mentioned techniques fail. That could explain the reduction of the mean operative time in their series. However, stone-free rate could be also reduced (93% vs 100%) for that. When compared to the results by PNL with endopyelotomy, the stone-free rate was equivalent (100%) and our obstruction relieving rate was higher (100% vs 90%) than that reported by Berkman and colleagues [13]. However, their cases included both primary and secondary UPJO while our series were all primary UPJO. Thus this difference could influence the success rate. Furthermore, their follow-up time was longer than ours. Therefore, long-term results of our series are awaited.

Our study also has some limitation. The use of the 2-camera system, allowing visualization and laparoscopic guidance of the rigid ureteroscopy, was required. However, this demand can be easily achieved in most medical centers.
